# Community and health workers perspectives on barriers to diabetes and hypertension screening in North Eastern India: a qualitative study

**DOI:** 10.1186/s12913-026-14435-z

**Published:** 2026-04-03

**Authors:** Balaplielad Warlarpih, Melari Shisha Nongrum, Baldeep K. Dhaliwal, YounJung Na, Preet Verma, Tiameren Jamir, Krishna D. Rao, Sandra Albert, Svea Closser

**Affiliations:** 1 Indian Institute of Public Health Shillong, Meghalaya, India; 2 Department of International Health, Johns Hopkins Bloomberg School of Public Health, Baltimore, MD USA; 3 International Vaccine Access Center, Johns Hopkins Bloomberg School of Public Health, Baltimore, MD USA; 4 Centre for Community Medicine, All India Institute of Medical Sciences, New Delhi, India

**Keywords:** NCD, NCD screening, Hypertension, Diabetes, Meghalaya

## Abstract

Non-communicable diseases (NCDs) such as diabetes and hypertension are growing global health concerns, including in India. Despite the launch of the National Programme for Cancer, Diabetes, Cardiovascular Diseases, and Stroke in 2010, screening uptake for diabetes and hypertension remains low in Meghalaya, a state in northeast India. We conducted a qualitative study with 52 participants including healthcare providers, frontline workers, community members, and Village Health Council members (VHC) to document barriers of uptake of diabetes and hypertension screening services. Health workers were selected from a sample of Health and Wellness Centres (HWCs) and the VHCs were drawn from the HWC catchment villages with active engagement in health activities. The research involved seven in-depth interviews and six focus group discussions across three blocks in West Garo Hills District. Data were transcribed, translated into English, uploaded into Taguette, and thematically analyzed. Our analysis identified several barriers to hypertension and diabetes screening across levels of the socio-ecological model. At the individual level, the cause and prevention of diabetes and hypertension were often associated primarily with diet. Work obligations, fear of diagnosis, and concern about long-term treatment discouraged participation in screening. Interpersonally, family members – particularly elders – played a key role in determining participation in screening. At the community level, limited involvement of VHCs and a preference for traditional medicine constrained screening efforts. At the policy level, a high village-to-HWC ratio, workforce vacancies and limited training created systemic barriers to screening. These same factors point to opportunities through intervention: providing screening and care that is reliable and easy to access, reaching out to trusted community messengers, and reducing logistical barriers to screening could all be effective strategies. These findings highlight the need for multipronged strategies that address individual, community, and systemic factors to enhance screening for early detection and management of NCDs in Meghalaya.

**Supplementary Information:**

The online version contains supplementary material available at 10.1186/s12913-026-14435-z.

## Background

Globally, Non-Communicable Diseases (NCDs) pose a major threat to public health, causing approximately 41 million deaths annually – 86% of which occur in low- and middle-income countries [1]. India is undergoing an epidemiological transition, with a rising dual burden of both infectious and non-communicable diseases.

Currently, NCDs account for 63% of all deaths in India, with cardiovascular diseases, cancers, chronic respiratory diseases, and diabetes being the primary contributors to NCD-related morbidity and mortality [2, 3]. Once an urban-specific issue, NCDs are rapidly increasing in rural areas [4, 5] driven by lifestyle shifts like poor diets, limited physical activity, and tobacco use [6]. NCDs also intensify poverty and inequality through increased out-of-pocket health expenditures, income loss, and financial insecurity [2]. In India, out-of-pocket expenses account for 58.7% of total health expenditures, a significant proportion is primarily due to NCD treatment [7].

The North-Eastern Region of India (NER) – comprising eight states and over 200 indigenous communities [8] – bear a disproportionately high burden of NCDs [9]. NCDs account for 66% of deaths in the region [10], with diabetes and hypertension prevalence estimated in the NER at 13.56% [11] and 21% [12] respectively, with diabetes exceeding the national average of 9.6% [13] and hypertension being slightly lower than the national average of 31.1% [14]. In Meghalaya specifically, a state in the NER, diabetes and hypertension prevalence stand at 15.6% and 24.3% respectively [15, 16].

In 2010, the Government of India launched the National Programme for Prevention and Control of Cancer, Diabetes, Cardiovascular Diseases and Stroke (NPCDCS). As part of this initiative, Accredited Social Health Activist (ASHAs) – India’s frontline cadre of community health workers – identify high risk individuals over 30 years old for NCD screening, using a Community Based Assessment Checklist (CBAC). Those scoring 4 or more on the CBAC are referred to the Health and Wellness Centres (HWCs) for further evaluation from Mid-Level Health Providers (MHLPs) and Auxiliary Nurse Midwives (ANMs) [17]. Screenings may also be conducted at HWCs and Village Health Nutrition Days (VHNDs) without a prior CBAC score. Screening and early detection is critical; untreated hypertension can lead to heart disease and stroke [18], and unmanaged diabetes can cause complications such as kidney failure, blindness, and amputations [19].

Despite efforts, screening rates in Meghalaya remain low. According to the National Cancer Registry Programme (NCRP) Report (2020), only 22.2% of eligible adults have been screened for diabetes [20], compared to the national average of 27.5% [21]. Hypertension screening stands at 42.5% [20], far below the national average of 70.5% [22]. Prior research in Jaintia Hills, Meghalaya cited systemic challenges such as workforce shortages, limited training, inadequate supplies, and social factors, highlighting barriers to the effective implementation of the NPCDCS [23]. However, this study focused only on one district and perspectives were mainly from the health system.

To address this gap, our study focuses on the Garo community – an Indigenous (Scheduled Tribe) community in the West Garo Hills District of Meghalaya. This paper explores both community and health system factors influencing the low uptake of diabetes and hypertension screening in the region, drawing from the perspectives of both health workers and community members.

## Methods

The study was conducted in the West Garo Hills District in Meghalaya – a region characterized by hilly terrain, remote villages with dispersed population. Research was carried out in three administrative blocks: Tikrikilla, Salsella, and Gambegre. These blocks were selected by the District Medical and Health Officer. Within each block, one Health and Wellness Centre (HWC) serving approximately 3,000 residents was purposively selected based on active community engagement and accessibility. From each HWC, the Village Health Councils were from the HWC catchment villages with active engagement in health activities.

The study included 52 participants: 8 healthcare providers (MLHPs and ANMs), 2 frontline health workers (ASHAs), 28 community members, and 14 VHC members. We conducted seven in-depth interviews (IDIs) and 6 focus group discussions (FGDs) with 3–11 participants in each FGD (Table 1). Data collection was facilitated in the participants’ home, village hall or HWC. All but one interview with a healthcare provider – which was conducted by phone – were held in person.

**Table 1:** Participant details

Sl no.	participant details	Gender		IDI	FGD
		Male	Female		
1	VHC members	7	7	0	2
2.	Youth	11	0	0	1
3.	Community members	13	4	4	1
4.	Healthcare providers	1	7	1	2
5.	Frontline health workers	0	2	2	0

Before beginning data collection, researchers explained the study’s purpose, participants’ rights, including confidentiality and voluntary participation. Participants were provided with a participant information sheet detailing the study objectives and their rights. Written informed consent was obtained from all participants.

The interview guide was developed specifically for this study based on literature review and expert inputs. The English version of the interview guide is provided as a supplementary file 1. The interview guide focused on several domains: current HWC service delivery, local understandings of hypertension and diabetes, services provided through NCD clinics, perceived barriers to screening, training and capacity building needs, and the use of technology. The guide was refined iteratively through daily debriefings during fieldwork to address emerging data gaps. Interviews and FGDs were audio-recorded, lasting between 30 and 100 min, and supplemented by field notes. The data collection was conducted in June 2024 by three female researchers with backgrounds in social work and public health, supported by Garo-speaking translators. All team members were trained in qualitative methods and research ethics prior to the data collection process.

Audio recordings were transcribed verbatim, initially into Garo and subsequently into English by two research team members. A third member independently reviewed all transcripts for accuracy. An inductive approach was used to code initial transcripts and develop a code book which was then used to code the remaining transcripts using Taguette. Thematic analysis was conducted to identify patterns and themes, using a collaborative process, with cross-checking by other researchers to ensure consistency and reliability of coding.

Community members and frontline health workers were not formally involved in the development of the topic guide or in the coding and analysis process. However, the research team engaged iteratively with participants and local stakeholders during data collection. The absence of formal community involvement in data analysis is acknowledged as a limitation of the study.

This study was conducted in accordance with the principles of the Declaration of Helsinki. The Institutional Review Board of the Johns Hopkins Bloomberg School of Public Health determined that the study was exempt from human subjects research oversight (IRB #4871).

## Results

**Fig. 1:**
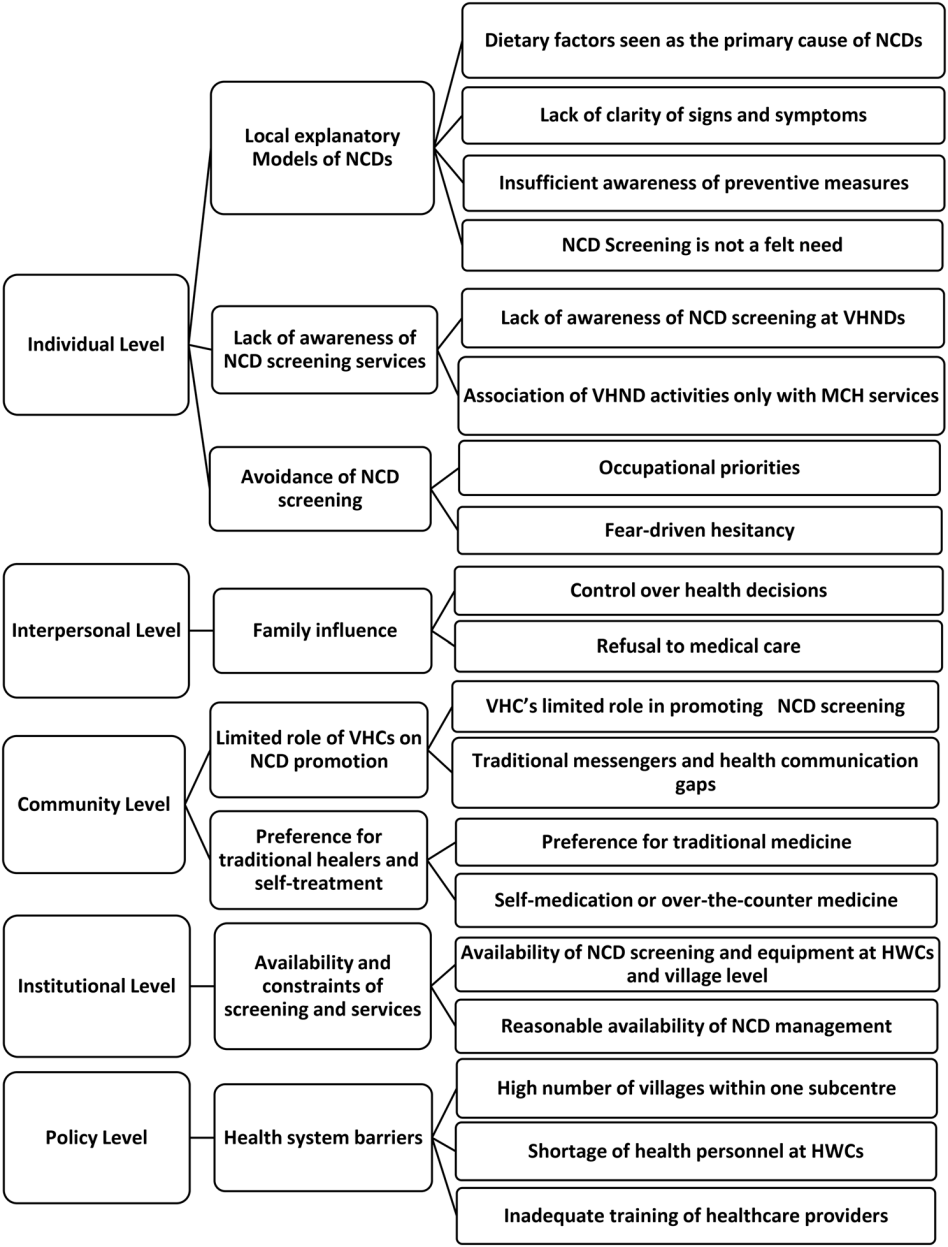
Socio-ecological framework explaining determinants of low participation in screening for hypertension and diabetes

**Fig. 2:**
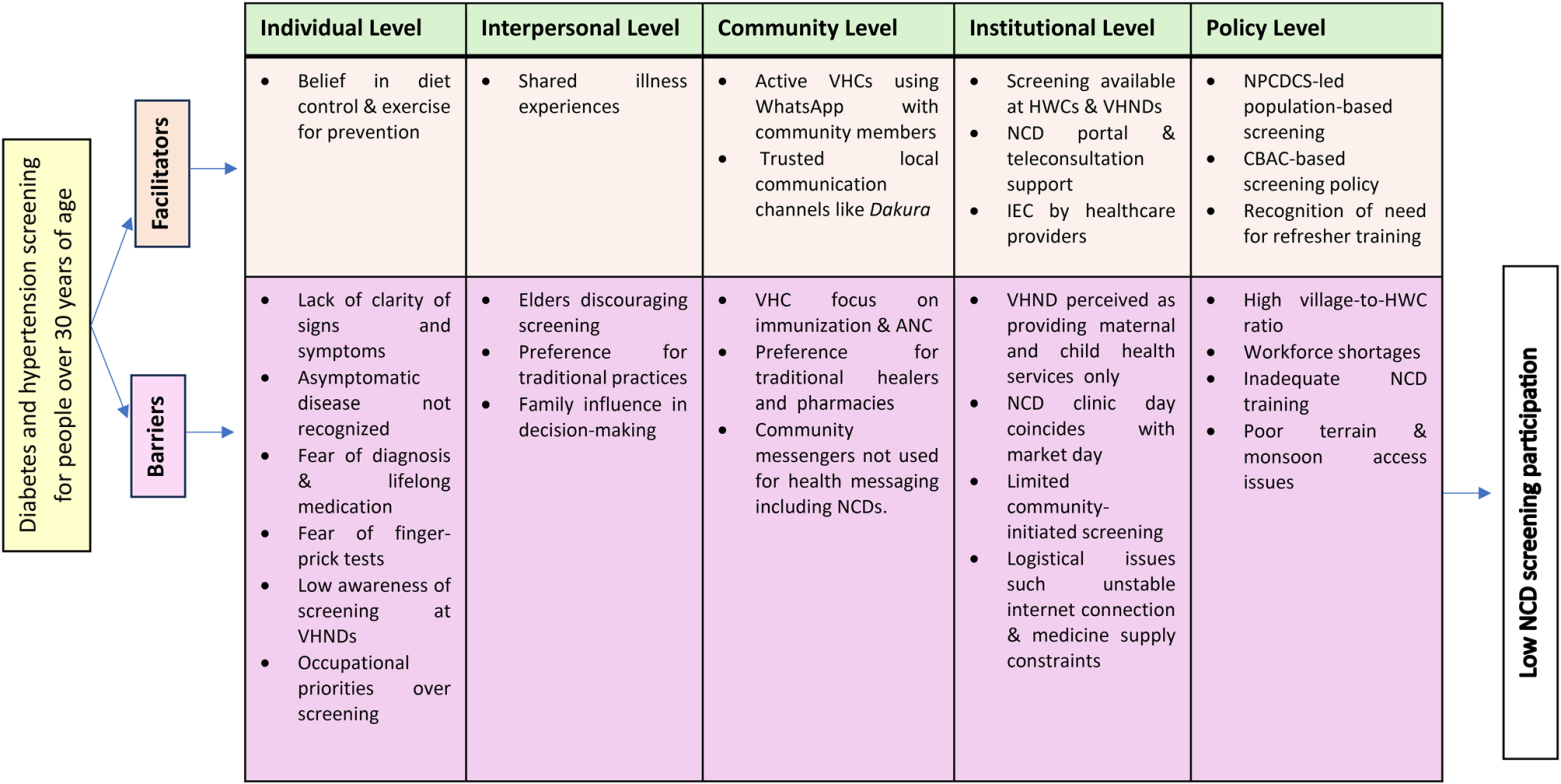
Socio-ecological barriers and facilitators influencing NCD screening participation

The findings were organized using the Socio-Ecological Model (SEM) [24], which highlights how health behaviors were shaped by and influence multiple levels of the broader social environment. It specifically considered interconnected influences, including personal characteristics, social networks, institutional structures, community resources, and policy-level regulations [25]. Building on this framework, we categorized barriers and facilitators to diabetes and hypertension screening across five thematic levels: (a) individual, (b) interpersonal, (c) community, (d) institutional, and (e) policy (see Figs. 1 and 2). These levels were deeply interconnected – individual behavior was continuously shaped by and reflective of broader social and systemic structures.

Participants were identified by anonymized codes, with ‘Healthcare provider’ referring to Auxiliary Nurse Midwifes (ANMs) and Mid-Level Health Providers (MLHPs), and ‘Frontline health worker’ referring to community health workers such as Accredited Social Health Activist (ASHAs).

At the individual level, respondents perceived the cause and prevention of diabetes and hypertension as linked to diets, often avoided diabetes and hypertension screenings, and were unfamiliar with VHND activities. At the interpersonal level, health seeking decisions were heavily influenced by families. At the community level, hypertension and diabetes screening was not a high priority, and many people preferred alternative treatment techniques. At health facilities, insufficient training and workforce vacancies coupled with a high village to HWC ratio impacted the monthly visits to the villages.

### Individual level

#### Local explanatory models of NCDs

Participants commonly referred to diabetes as “chini” (sugar) and hypertension as “pressure” or “blood pressure.” These terms do not necessarily map neatly into the concepts of diabetes and hypertension used in biomedicine. Both conditions were widely believed to be caused by diet by both community members and frontline workers.

Community members perceived these foods to contain chemicals due to the introduction of modern agricultural methods where *‘chemicals’* are used in the chicken feed, and chemical fertilizer and pesticides are used for growing vegetables; these were associated with hypertension. For example, a community member said that the consumption of broiler chicken (chicken raised with intensive farming methods for meat production) and vegetables may be a cause for hypertension. Additionally, excessive intake of salt during meals and *“having too much blood in the body”* was associated with the person having hypertension.

The frontline health workers also emphasized diet as the cause of hypertension and diabetes. For example, one commented that that the cause of hypertension was due to *“eating food like fats and those foods which have iron like eggs and meat.”* The intake of ‘sweets’ of any form was commonly perceived by community members to be a cause of diabetes.

Both the community members and frontline health workers believed that dietary changes could help prevent hypertension and diabetes. One community member reported “*If we maintain our diet and reduce the sugar intake*,* we can prevent it”.* Frontline health workers largely believed that dietary adjustments such as *“eating bitter vegetables and less potatoes*” would prevent diabetes. Health care providers additionally emphasized that regular exercise would normalize blood pressure and diabetes.

Health information was often passed through personal experiences and informal networks such as neighbors and relatives. Community members commonly associated hypertension with headaches, body pain, fainting, and yellowish eyes—though some of these may not be direct symptoms from the perspective of biomedicine. Similarly, diabetes was linked to body pain, tingling sensations, chest pain, shortness of breath, swelling, fatigue, and itchiness. For example, one community member commented, *“My father also has diabetes; he usually complains about shortness of breath and fatigue”*. The healthcare providers and VHC members also reiterated that community members sought medical checkups when they experienced dizziness, assuming it might be related to hypertension. *“Usually when people have dizziness we suspect ‘sugar’ or ‘pressure’ and we go for a checkup. For hypertension*,* we get dizzy and fatigue but for diabetes*,* we get fatigue.”*

Still, community members did not always seek care when they had these symptoms. They identified other symptoms or illnesses such as *stomach pain*,* fever*,* chickenpox*,* and measles –* as reasons to seek care, rather than these symptoms. They also did not discuss early detection of hypertension and diabetes in people without obvious signs or symptoms.

#### Lack of awareness of NCD screening services

Most community members were unaware that diabetes and hypertension screenings were available during the Village Health and Nutrition Days (VHNDs). VHND were largely perceived as services for pregnant women and children, limiting broader community participation.

A healthcare provider commented, *“They think that the healthcare providers are coming to check pregnant mothers and children*,* that is why most of the villagers do not come.”*

Only a handful of eligible individuals (age 30 years and above) attended and participated in the diabetes and hypertension screenings during VHNDs. As one VHC member shared: *“People don’t know that they are screening for sugar and pressure during VHNDs. If people knew about it*,* they would go for the check-up.”*

Although healthcare providers try to enhance awareness by, as one described it, “*Using posters and providing oral presentations to share information about NCDs during VHNDs”*, these efforts primarily reach only mothers as they constitute the majority of VHND attendees. Frontline workers also conduct home visits to encourage people to participate in diabetes and hypertension screenings. But even with these efforts, many eligible adults remained unaware of screening availability as these services tended to be overshadowed by immunizations and ANC check-ups.

#### Avoidance of NCD screening

Community members often prioritized agricultural labor or daily wage work over attending screenings during work hours. A frontline health worker confirmed this, saying *“Even though they know a VHND is happening in their village*,* some individuals prioritize their daily work and choose not to attend.”*

Health workers said that some community members expressed fear of medical procedures like finger-pricks test for blood sugar. *“Actually*,* they are scared of needle pricking for checking sugar*,*” one* healthcare provider said. Some said community members often felt worried and reluctant to discover health issues like hypertension and diabetes, largely due to the perceived burden of long-term treatment. A healthcare provider commented “*Some of them say*,* ‘Oh*,* If I get hypertension*,* then I have to take medicine. I have to go again and again and again to the CHC.”*

Additionally, some community members refused screenings, saying they had no illness and did not feel the need to screen. Health care providers also reported that, even with sustained IEC (Information, Education, Communication) efforts, there was limited uptake. *“After giving so much IEC they don’t want to accept*,*”* a healthcare provider commented. However, IEC efforts, which primarily reached women and excluded other groups such as men and the elderly, which contributed to low screening services among the broader population.

### Interpersonal level

#### Family influence

Healthcare providers emphasized the role of elders – particularly parents and grandparents- in influencing health decisions. If elders discouraged screening, other family members often complied. This was also evident in other services such as maternal care, institutional deliveries, and immunizations.

Healthcare providers stated that this attitude from elders stemmed from the traditional beliefs and prior positive experiences of elders delivering children at home or having unvaccinated children. One healthcare provider reported, *“Some depend on family members. If their mother says don’t go*,* that means they won’t come.”*

Healthcare providers also reported that such households preferred traditional healers over institutional care, further impacting screening participation.

### Community level

#### Limited role of VHCs in NCD promotion

While some VHCs actively shared health information through WhatsApp groups, most focused on maternal and child health. In certain villages, this approach improved participation in screenings during VHNDs, with eligible individuals coming forward for screening. *“Everyone does come whether it is for testing or program because we have a WhatsApp group*,* we will inform there*,” one healthcare provider asserted. However, many community members reported being unaware that hypertension and diabetes screenings were part of VHNDs.

While VHC members reported that one of their main activities was conducting home visits, these home visits were typically geared towards immunization and maternal and child related issues, as opposed to NCD screenings. None of the VHCs reported conducting home visits to encourage NCD screenings.

In many villages in the Garo communities, a traditional community messenger (“*Dakura*”) appointed by the local head (‘*Nokma’)* would often inform community members about important events or meetings in the community, such as *Nokma*-initiated village meetings. However, the *Dakura* was not asked by the *Nokma* to convey NCD-related services or health-related messages, as these were seen as the ASHA’s responsibility.

#### Preference for traditional healers and self-treatment

In addition to formal healthcare services, community members often relied on a mix of traditional, self-managed, and over-the-counter treatment approaches. Traditional healers, (*“Oja”* in Garo) are seen as an important source of care for ailments ranging from seizures, hypertension, malaria, to jaundice. Community members would seek care with traditional providers based on the type of illness. One member reported, *“For dysentery*,* we go to the traditional healer*,* and for fever*,* we go to the pharmacy.”* Additionally, many community members preferred traditional healers or birth attendants for childbirth, opting for culturally-rooted practices over institutional care.

Self-management and the use of over-the-counter remedies were both mentioned as common by community members. Pharmacies often served as the first point of contact for immediate healthcare needs due to their accessibility. *“We have a pharmacy near our village. I go there only*,*”* one participant reported.

The community also used herbal remedies for conditions like hypertension. As one community member stated, *“We don’t have prevention for sugar*,* but pressure can be prevented by eating a plant called Dongam.*” Another reported similar benefits from sour fruits, with one participant reporting, *“Once I ate pomelo fruit and checked*,* that time my pressure was back to normal.”*

### Institutional level

#### Availability and constraints of screening services

Healthcare providers reported the availability of screening services at HWCs, including a dedicated NCD clinic day (usually Tuesdays). One reported, *“We do screenings for hypertension*,* diabetes*,* and cancer at the HWC whenever the patients visit. We also have a dedicated NCD clinic on Tuesdays. Screenings are also conducted during field visits (VHNDs).”*

The healthcare providers also mentioned that designated NCD Clinic Day coincides with the market day, saying, *“it’s a weekly market day actually*,* so we are not getting many patients.”*

These screenings were available for community members aged 30 + or those with high CBAC scores, as well as individuals who had not undergone a prior CBAC screening. The screenings were available throughout the week at HWCs and not limited to NCD clinic days. However, none of the community members explicitly stated that they went to the health centres for H & D screening.

While all interviewed healthcare providers reported no shortages of screening equipment, a community member highlighted occasional medication shortages at HWCs, *“Sometimes they run out of medicines.”*

The healthcare providers reported that they managed hypertension and diabetes by uploading results to the NCD portal and initiating teleconsultations for diagnosis, tests, and monitoring over a month. However, internet connectivity issues hampered teleconsultation workflow with healthcare providers resorting to phone calls to Primary Health Centre (PHC) medical officers instead. One said, *“Sometimes we call the medical officer for NCD consultation.”*

### Policy level

#### Health system barriers

A key barrier to accessing NCD services was the large number of villages assigned to some HWCs which catered to small and sparsely populated villages. A health worker commented, *“Because we have 30 villages under our HWC*,* we cannot go for field visits*,* and the people*,* they don’t come to the health centre.”*

The high number of villages each HWC is required to cover made it difficult to conduct monthly VHNDs in each village, despite their importance for building trust and encouraging community participation in health programs.

Further, staffing shortages due to hiring delays exacerbated this issue. In some cases, a single ANM handled all responsibilities—including immunizations and NCD screenings. One healthcare provider reported, *“When there was an MLHP*,* she used to do diabetes and hypertension screening*,* and I used to do immunization*,* but now since I am alone*,* I am doing both.”*

In addition to immunization and screening, healthcare providers were also responsible for follow-up care of high-risk patients, which increased their workload significantly. Healthcare providers, particularly ANMs, felt underprepared to manage NCD services, and they reported limited training opportunities, with last NCD related training, occurring four years ago. The need for regular refresher training was emphasized, with one health worker commenting, *“I think it will be better to get the training because it’s better to update again and again.”*

In addition, while healthcare providers faced challenges to reach villages for VHNDs due to poor roads, long distances, and hilly terrain, these challenges worsened during the monsoon season when rising water levels make shortcuts inaccessible and roads nearly unusable. One commented, *“During monsoon the road conditions are bad so we have to walk as we cannot just cancel the date fixed for VHND. Sometimes it is difficult to get a bike so we have to walk 1 hour which usually takes 30 min by bike.”*

### Cross-cutting barriers and opportunities for action

Taken together, these findings illustrate how individual beliefs, household dynamics, community norms, institutional capacity, and policy constraints intersect to shape participation in hypertension and diabetes screening. Gaps in awareness, culturally rooted health practices, competing livelihood demands, and logistical system challenges compound one another. These barriers do not operate in silos; they reinforce each other in ways that quietly discourage engagement with preventive care.

Yet, within each level there lies opportunities for change. By addressing these barriers in a coordinated and context-sensitive manner, there is significant potential to improve screening participation and strengthen trust in the health system. The insights from this study can serve as a foundation for more inclusive, community-anchored strategies to advance NCD prevention in Meghalaya, in India, and beyond.

## Discussion

Our findings show that low participation in diabetes and hypertension screening in West Garo Hills is shaped by multi-level factors, including low awareness of the importance of screening, strong family influence, competing priorities within the community, and systemic health system constraints such as staff shortages and insufficient training. These results align with studies from other low-and-middle-income countries that identify similar barriers: workforce deficits, limited diagnostic capacity, poor awareness, stigma, and inadequate focus on prevention [26–28]. In India, these challenges are compounded by unfilled positions, undertrained staff, inconsistent medicine supplies, and under-resourced laboratory services [29] as well as poor awareness and differing conceptions of these conditions between communities and care providers [30].

### Addressing barriers at the individual and household level

A lack of understanding about the importance of early detection emerged as a key barrier to screening in West Garo Hills, aligning with findings from studies in northern and western India [31, 32]. Participants often linked hypertension and diabetes solely to dietary habits and sought to manage risk through dietary changes that may not necessarily be effective. Since the conditions are typically asymptomatic, many community members gauged their health based on how they felt, which could delay diagnosis and treatment. Similar behaviors were observed in studies from Ethiopia [33] where risky behavior such as tobacco and alcohol consumption– prevalent in Meghalaya — were often overlooked. Similarly in studies from Kenya and South India, individuals with limited knowledge of NCDs often deprioritized diabetes and hypertension screening [34] delayed care seeking [35, 36].

Evidence suggests there are strategies that can help in underserved areas. Storytelling and testimonials from people who have benefited from early detection through screenings can be beneficial [37], as can advertising screening through local gatherings and social media platforms, and providing opportunities for screening in the home [38]. Community institutions like Self Help Groups (SHGs) [39] and frontline health workers [40] can also be mobilized to raise awareness, conduct disease surveillance, and provide resources for preventive outreach. Other outreach interventions such as telephone reminders, personalized invitation letters from medical practitioners, and scheduled appointments – instead of broad, open calls– have improved uptake elsewhere and could also be adapted [41]. Health fairs have also been shown to enhance awareness and participation in screening programs in underserved communities [42]. Given that agricultural work and daily wage labor often take precedence over preventive health services, screening strategies in West Garo Hills may need to extend beyond fixed VHND schedules. House-to-house screening for older adults and men, particularly during early morning hours or agricultural off-seasons, could help address work-related constraints and reduce missed opportunities for early detection. Incorporating blood pressure and blood glucose screening in Mahatma Gandhi National Rural Employment Guarantee Scheme (MGNREGS) worksites during working hours may improve NCD screening coverage among underserved populations.

### Addressing interpersonal and cultural barriers

Our study found that family members – particularly elders – influence health decisions, often discouraging screening or favoring traditional healing practices. This aligns with previous research on bone fractures among the Garo tribe of Meghalaya [43] and cancer treatment delays in Meghalaya [44]. However, families can also be powerful allies: family support is associated with better adherence to diets and follow-up care among people with NCDs [45, 46] highlighting the importance of engaging with the elders in the family. As elders strongly influence household health decisions, screening initiatives should engage elders and influential community members to legitimize screening and enhance community participation. Such efforts should be accompanied by clear, locally tailored messaging on the role of diet, physical activity, weight management, and avoidance of tobacco products in preventing hypertension and diabetes, along with guidance on when to seek formal care. Traditional community messengers, such as the *Dakura*, could also be engaged to announce VHND dates and explicitly mention NCD screening services. Engaging traditional healers—who are respected —may help bridge the gap [47, 48]. Traditional healers could be sensitized to recognize NCD risk and encourage referrals to HWCs, positioning them as referral allies rather than alternatives to biomedical services. In other global contexts, traditional healers have played a role in promoting screening and encouraging biomedical care [49, 50].

### Addressing barriers at the community level

At the community level, trusted leaders – including village elders, village headmen *(Rangbah Shnong/Nokma)*, religious leaders, and traditional healers – can play a pivotal role in increasing awareness and participation in screening. VHCs, which currently focus mostly on maternal and child health, could be sensitized and trained to incorporate diabetes and hypertension into their health agenda. This approach aligns with research from Meghalaya showing that increased community awareness contributed to higher participation in malaria prevention efforts [51]. During the COVID-19 pandemic, traditional village councils were critical in implementing local public health responses [8], reinforcing their potential role in NCD-related outreach.

In addition to awareness generation, community-based approaches to prevention and self-monitoring may be particularly relevant in West Garo Hills. Training one adult member from each household to measure blood pressure using digital BP monitors and blood sugar using glucometer could promote early detection and reduce dependence on facility-based screening alone. Making a small number of BP machines and glucometers available at the village level—managed through VHCs or self-help groups—may enable regular monitoring as part of routine health practices.

### Addressing health system constraints

Health system constraints – particularly insufficient staffing where healthcare workers are often tasked with multiple responsibilities – has had major effects on screening participation in our study. Similar challenges have been reported in Kenya, Nepal, and North India [52–54]. When one HWC is responsible for 30 or more dispersed villages, holding regular VHNDs becomes difficult. Our findings suggest a need for the state to reconsider current population and geographic coverage guidelines for HWCs in remote and sparsely populated regions.

Additionally, health workers voiced a need for more frequent training. This finding resonates with other contexts [55] and evidence from other areas have shown that steps can be taken to improve this. Evidence from the Philippines demonstrated that programs such as the Blood Pressure Measurement Training Program, significantly improved provider knowledge and screening delivery [56].

The multi-level factors outlined in the Social-Ecological Model (SEM) are not isolated – they reinforce and compound each other. Individual factors are deeply shaped by social factors, and by experiences with the health system. A fragmented response is unlikely to succeed. Instead, coordinated, multi-level interventions are needed to improve screening uptake and reduce the burden of NCDs.

## Conclusions

This study contributes to a growing body of research that emphasizes the complexity of NCD screening in marginalized and Indigenous communities. While many previous studies focus on systemic barriers, our findings illustrate the importance of understanding how social, cultural, and institutional forces interact to shape health behavior, and the need to collectively consider these barriers while developing interventions. Addressing these dynamics holistically, in collaboration with the communities affected, will be essential to advancing NCD prevention and improving public health outcomes in Meghalaya and beyond.

## Supplementary Information

Below is the link to the electronic supplementary material.

Supplementary Material 1

## Data Availability

The qualitative datasets (interviews and focus group transcripts) generated and analyzed during the current study are not publicly available to protect participant confidentiality. De-identified excerpts that support the findings are available from the corresponding author upon reasonable request.

## Abbreviations

NCDs: Non-communicable Diseases
NER: North-Eastern Region
NPCDCS: National Programme for Prevention and Control of Cancer, Diabetes, Cardiovascular Disease and Stroke
ASHA: Accredited Social Health Activist
CBAC: Community Based Assessment Checklist
HWC: Health and Wellness Centre
MLHP: Mid-Level Health Provider
ANM: Auxiliary Nurse Midwife
VHND: Village Health Nutrition Day
NCRP: National Cancer Registry Programme
VHC: Village Health Council
IDI: In-Depth Interview
FGD: Focus Group Discussion
SEM: Socio-Ecological Model
IRB: Institutional Review Board
MGNREGS: Mahatma Gandhi National Rural Employment Guarantee Scheme
