# Supplementary material for: Community and health workers perspectives on barriers to diabetes and hypertension screening in North Eastern India: a qualitative study
Source: BMC Health Serv Res. 2026 Apr 3;26:696. doi: 10.1186/s12913-026-14435-z (PMC13173876; doi:10.1186/s12913-026-14435-z)
Supplement: Supplementary file 1 — Supplementary Material 1 [file 12913_2026_14435_MOESM1_ESM.docx]

Interview guide:

1. In depth interviews (IDI) with staff of hwc

| Topic | Probes |
| --- | --- |
| COMMUNITY HEALTH OFFICER/ MEDICAL OFFICER/STAFF OF HWC | |
| Introduction | Introduction  ·   Name  ·   Designation  ·   Years of experience  ·   Duration of engagement in the particular centre  General conversation  ·   Current roles and responsibilities |
| Information on service delivery | Can you please tell us on the type of services provided at your facility related to ncds?  ·       Diagnostic  ·       Prognostic  ·       Therapeutic  ·       Outreach activity  Can you please explain us the process of service delivery in your health center? Especially Hypertension and Diabetes  ·       Who is in charge?  ·       Who supervises the delivery of the services?  ·      What is your role in delivery of the services in your center? ( |
| Assessment of current Services | Can you describe the strengths and weaknesses of services delivered at your facility? |
| Exploration on newer service packages | Can you tell us on newer services packages provided under HWC?  Tell us your thoughts on the newer services packages  ·   Demand in the community  ·   Acceptance in the community  ·   Service delivery- in terms of availability of services, availability of HR for delivery of the services  ·   Utilisation of services |
| Concept of ncds | What does the community understand by NCD (diabetes and hypertension)? (probe: local term, symptoms that trigger people to seek treatment, health seeking behaviour, preventive steps taken by community) |
| Needs and Gaps in services delivered at hwcs | What are the gaps or shortcomings that you think are present in the NCD services provided by HWC  The areas that require improvement or expansion   - What are the needs of the local population that are not adequately addressed by the current service offerings |
| Barriers to Implementation: | What are the potential barriers in providing NCD (hypertension and diabetes) services?, (Probes : financial constraints, lack of resources, infrastructure limitations, or resistance from staff or patients)  Can you elaborate any specific challenge related to providing services for hypertension and diabetes?  What are few strategies that you believe could overcome these barriers?  Can you demonstrate that some of these strategies have helped in overcoming these barriers? |
| Training and Capacity Building | Can you please tell us about the training you have received or attended in the last 1 year? (related to NCD services) (Probe: who attended, where, when, number of days, gaps in skills that exists after training)  Do you think that the services you provide now have improved after the training? (probe: identify any gaps that still exist)  Can you explain if you think training or skill development is required for health care providers to deliver these services effectively.  Can you explain how the newer training could be delivered to the staff at your facility? (Probe: preferred manner of the training, reasons for such preferences) |
| Integration of technology | What are your thoughts on role of technology in improving the service delivery such as telemedicine or digital data collection or online portals for data entry.  Are you able to link your work to the CBAC information or family folder that has been collected? |
| Way forward | What are your thoughts in the long term sustainability of ncds services  Mention some strategies that can be helpful to ensure that newer services remain available or effective  Can you prioritise the services provided at your facility based on feasibility and potential impact. |

1. Focus Group Discussions (FGD) with community members

| Topic | Probes |
| --- | --- |
| Introduction | Introduction  ·   Name  ·   Gender  ·   Age  ·   Educational qualification  ·   Occupation  ·   Duration of stay in the current locality  General conversation  ·   Experience with health care services  ·   Involvement in the local HWC or local administration (Village Council) |
| Overview of Current Services | Can you tell us about the current services provided at the HWC?  What are your experiences with the existing services  ·    What you find helpful?  ·    Any areas you believe need improvement (HR, Logistics, Infrastructure, Finance, Etc)  Can you tell us about the NCD services provided under HWC?  Can you tell us if these NCD services address the needs of the community better than before? |
| Concept of NCD | What is the general understanding about NCD?  (Probe: diabetes and hypertension – local term, perceived susceptibility, severity, symptoms that trigger people to seek treatment, health seeking behaviour -home remedies, self-management, traditional medicine, health facility, preventive steps taken by community) |
| Health needs of the community | What are the common ailments in the community that needs the HWC’s attention? (Probe: Hypertension, diabetes)  Can you tell us about any specific health concerns, common ailments or services that are not adequately addressed by the current health care services? |
| Accessibility and Barriers | Please tell us about the accessibility of ncds services at the HWC.  Can you tell us about any barriers villagers face in accessing ncds such as distance, transportation, cost or cultural factors? |
| Community Engagement | Can you tell us about various platforms which could be utilised for community engagement towards rolling out of NCD services from the hwcs?  What do you think about community engagement in decision making or involving the local leaders could be beneficial in improving the reach or utilisation of NCD services delivered at HWC?  What are the barriers incase this is not happening? |
| Awareness and Education | What are the most effective ways to inform villagers about NCD services and educate them about their benefits.  What could be potential strategies for raising awareness and addressing any concerns or misconceptions. |
| Preference and acceptance to services | Are there any cultural beliefs related to NCD services?  How cultural beliefs and practices might impact the acceptance of newer services such as NCD services  Can you share your thoughts on which specific services or types of care that you believe would be most valuable for the community.  What type of services that would make the most significant positive impact on villagers' health? |
| Way forward | Can you describe the ways in which the new healthcare services can benefit the community on a long term basis?  Any other suggestions or comments. |

1. IDIs with Community Members

| Topic | Probes |
| --- | --- |
| Introduction | Introduction  ·   Name  ·   Gender  ·   Age  ·   Educational qualification  ·   Occupation  ·   Duration of stay in the current locality  General conversation  ·   Experience and access to health care services  · |
| Overview of Current Services | Can you tell us about the current services provided at the HWC?  What are your experiences with the existing services   - What you find helpful? Can you demonstrate or give an example of when you found the services helpful? |
| Concept of NCD | What is your understanding about NCD?  (Probe: diabetes and hypertension – local term, symptoms that trigger you to seek treatment, health seeking behaviour, home remedies, self-management, traditional medicine, health facility, preventive steps) |
| Health needs of the community | What are the common ailments that you need the HWC’s attention? (Probe: Hypertension, diabetes)  Can you tell us about any specific health concerns, common ailments or services that are not adequately addressed by the current health facility? |
| Accessibility and Barriers | Have you accessed any NCD services? (Probe: experience of the service)  Are you facing accessing ncds such as distance, transportation, cost or cultural factors (probe: cultural and social factors, beliefs ) |
| Community Engagement | Can you tell us about various platforms which could be utilised for community engagement towards rolling out of NCD services from the hwcs?  What do you think about community engagement in decision making or involving the local leaders could be beneficial in improving the reach or utilisation of NCD services delivered at HWC?  What are the barriers in case this is not happening? |
| Awareness and Education | What are the most effective ways to inform people in your community about NCD services and educate about their benefits.  What could be potential strategies for raising awareness and addressing any concerns or misconceptions. |
| Preference and acceptance to services | Are there any social factors or cultural beliefs related to NCD services?  How social factor cultural beliefs and practices might impact the acceptance of newer services such as NCD services?  Can you share your thoughts on which specific services or types of care that you believe would be most valuable for the community.  What type of services that would make the most significant positive impact on people’s health? |
| Way forward | Can you describe the ways in which the new healthcare services can benefit the community on a long term basis?  Any other suggestions or comments. |
